# Anti-tumor activity of selective inhibitor of nuclear export (SINE) compounds, is enhanced in non-Hodgkin lymphoma through combination with mTOR inhibitor and dexamethasone

**DOI:** 10.1016/j.canlet.2016.09.016

**Published:** 2016-09-28

**Authors:** Irfana Muqbil, Amro Aboukameel, Sivan Elloul, Robert Carlson, William Senapedis, Erkan Baloglu, Michael Kauffman, Sharon Shacham, Divaya Bhutani, Jeffrey Zonder, Asfar S. Azmi, Ramzi M. Mohammad

**Affiliations:** a Department of Oncology, Wayne State University, USA; b Karyopharm Therapeutics Inc, Newton, MA, USA

**Keywords:** NHL, Exportin-1, XPO1, CRM1, SINE, Selective inhibitors of nuclear export

## Abstract

In previous studies we demonstrated that targeting the nuclear exporter protein exportin-1 (CRM1/XPO1) by a selective inhibitor of nuclear export (SINE) compound is a viable therapeutic strategy against Non-Hodgkin Lymphoma (NHL). Our studies along with pre-clinical work from others led to the evaluation of the lead SINE compound, selinexor, in a phase 1 trial in patients with CLL or NHL (NCT02303392). Continuing our previous work, we studied combinations of selinexor-dexamethasone (DEX) and selinexor-everolimus (EVER) in NHL. Combination of selinexor with DEX or EVER resulted in enhanced cytotoxicity in WSU-DLCL2 and WSU-FSCCL cells which was consistent with enhanced apoptosis. Molecular analysis showed enhancement in the activation of apoptotic signaling and downregulation of XPO1. This enhancement is consistent with the mechanism of action of these drugs in that both selinexor and DEX antagonize NF-κB (p65) and mTOR (EVER target) is an XPO1 cargo protein. SINE compounds, KPT-251 and KPT-276, showed activities similar to CHOP (cyclophosphamide–hydroxydaunorubicin–oncovin–prednisone) regimen in subcutaneous and disseminated NHL xenograft models *in vivo*. In both animal models the anti-lymphoma activity of selinexor is enhanced through combination with DEX or EVER. The *in vivo* activity of selinexor and related SINE compounds relative to ‘standard of care’ treatment is consistent with the objective responses observed in Phase I NHL patients treated with selinexor. Our pre-clinical data provide a rational basis for testing these combinations in Phase II NHL trials.

## Introduction

Non-Hodgkin lymphoma (NHL) is a heterogeneous class of hematological malignancies that display a diverse range of biological phenotypes, clinical behaviors and prognoses [1]. Standard treatments for NHL involve anthracycline-based combination regimen comprising of cyclophosphamide, doxorubicin, vincristine and prednisolone (CHOP) [2]. Although addition of rituximab to this regimen (R–CHOP) has improved response rates (40–50%) [3], a substantial proportion of patients relapse, resulting in 3-year overall survival rates of ~30%. Relapsed lymphomas are often refractory to subsequent chemotherapy regimens and exhibit adaptive resistance to a wide variety of other anti-cancer drugs. The emergence of acquired chemoresistance thus poses a challenge in the clinic preventing the successful treatment of this relapsed disease.

Signaling by the PI3K/AKT/mTOR pathway is frequently deregulated in NHL, prompting evaluation of the rapamycin-analog (rapalog) or mTOR inhibitors in multiple clinical trials [4]. These rapalogs (e.g. everolimus or EVER) show activity as single agents and are an acceptable therapeutic option especially in relapsed/refractory mantle cell lymphoma. Response rates, however, are typically <50%, resulting in remissions that are neither complete nor durable. It is likely that PI3K/AKT/mTOR inhibition will find more prominent role in NHL therapy provided a superior combination of rapalogs with other novel therapies are identified that may help to bypass the heterogeneity driven resistance mechanism of this disease [5].

The glucocorticoid therapy, dexamethasone (DEX), is a featured treatment in majority of the combination regimens for advanced lymphomas [6]. While useful in clinical practice, patients taking this glucocorticoid (GCs) for extended periods often suffer from skeletal side effects including growth retardation in children and adolescents, and decreased bone quality in adults [7]. Nevertheless, the addition of DEX to CHOP, R–CHOP, or other platinum based combinations showed improvement of the overall survival in childhood and adult NHL. Its incorporation helped improve the management of toxicities associated with combination chemotherapeutic regimens [8]. However, these studies indicate that better management of DEX dosing, scheduling or combinations are needed.

In eukaryotic cells, the main mediator of protein export from the nucleus to the cytoplasm is the transporter, exportin 1 (XPO1), also known as chromosomal region maintenance 1 (CRM1) [9]. XPO1 is a member of the importin-β superfamily of nuclear export receptors called karyopherins, which can interact with leucine-rich nuclear export signals (NESs) [10]. Earlier studies have clearly demonstrated the role of XPO1 in promoting NHL cell growth. In these studies, XPO1 over-expression was shown to downregulate the tumor suppressor p27^Kip1^ thereby regulating cell proliferation. Large scale analysis of NHL patient data also points to a negative correlation between XPO1 over-expression and progression free/overall survival. Given the critical role of these nuclear exported molecules in proliferation and survival, XPO1 is certainly a valid therapeutic target for NHL. This is especially critical for tumor suppressor proteins (TSPs) and cell cycle regulators that must localize to the cell nucleus in order to properly function [11].

SINE compounds shown to specifically target cysteine 528 of XPO1 [12–14] can induce nuclear localization of TSPs and inhibit NHL cells at low nano-molar concentration *in vitro*. SINE compounds can also suppress growth of WSU-DLCL2 sub-cutaneous tumors in mice as well as enhance mouse life span in a WSU-FSCCL systemic model when compared to vehicle treatment [15]. These XPO1 inhibitors demonstrate comparable anti-lymphoma activity to that of a CHOP regimen used on lymphoma xenografts in mice. Our pre-clinical data along with others and the Phase I trial of the SINE compound selinexor (KPT-330) in advanced hematological malignancies (NCT01607892) led to a Phase I evaluation of selinexor in patients with CLL or NHL (NCT02303392). In this paper, we show that selinexor can enhance the activity of DEX and EVER both in NHL cell lines *in vitro* and *in vivo*. These results build the case for the potential clinical application of SINE-DEX and SINE-EVER for patients with resistant NHL.

## Materials and methods

WSU-FSCCL, representing follicular small cleaved cell lymphoma and WSU-DLCL2, representing diffuse large cell lymphoma were developed and characterized in our laboratory at Wayne State University [16–18]. SINE compounds selinexor, KPT-185, and KPT-301 (inactive analog) were provided by Karyopharm Therapeutics Inc (Newton, MA). Primary antibodies for PARP, Full length caspase were purchased from Cell Signaling (Danvers, MA USA). β-actin antibody and all secondary antibodies were obtained from Sigma (St. Louis, MO, USA).

### Cell growth inhibition determined by the trypan blue assay

Cells were seeded at a density of 2 × 10^5^ viable cells/mL in 24-well or 6-well culture plates (Costar, Cambridge, MA, USA), or 10-cm cell culture dishes (Corning Inc., Corning, NY, USA). All cells were maintained in RPMI 1640 medium supplemented with 10% fetal bovine serum (Hyclone Laboratories, Logan, UT, USA) and 1% penicillin-streptomycin (Invitrogen, Carlsbad, CA, USA), at 37 °C in a humidified incubator with 5% CO_2_. The number of viable cells was determined by a trypan blue exclusion test [trypan blue (0.4%), Sigma Chemical Co. St. Louis, MO, USA]. KPT SINE, DEX and EVER were added at indicated concentrations (0–150 nM) diluted from a 10 µM stock. The results were plotted as means ± SD of three separate experiments using three determinations per experiment for each experimental condition.

### Quantification of apoptosis by histone DNA enzyme-linked immunosorbent assay and annexin V-FITC assay

Cell apoptosis was detected using an Annexin V-FITC assay (Biovision, Danvers, MA, USA) and a Histone DNA ELISA Detection Kit (Roche, Life Sciences) according to the manufacturers' protocols. NHL cells were seeded as described previously and treated with SINE, DEX and EVER alone and in combination for 72 h. All procedures were performed according to our previously published protocols [15].

### Western blot analysis

Cells (1 × 10^6^) were grown in 6-well petri plates and exposed to indicated concentrations of SINE, DEX and EVER alone and in combination for 72 h followed by extraction of whole cell proteins for western blot analysis using previously described methods [15].

### Immunofluorescence assay for p65 cellular staining

For protein localization experiments, 1 × 10^6^ cells were grown in 24-well plates and exposed to selinexor, DEX and EVER alone and in combination at indicated concentrations for 24 h. At the end of the treatment the cells were mounted on glass slides using cytospin (2500 rpm for 10 s twice) followed by fixing with 10% paraformaldehyde for 20 min. The fixed slides were permeabilized using 0.5% Triton (Sigma, St Louis, USA) and were blocked in 0.2% BSA for 45 min. The slides were probed with primary (p65) and secondary antibodies (Alexa Fluor conjugated goat anti-rabbit). The slides were then dried and mounting medium was added; the slides were covered with coverslips before analysis under an inverted three-color (DAPI, GFP and RFP) fluorescent microscope.

### RNA isolation and RT-PCR

WSU-DLCL2 cells were grown at density of 200,000 cells per well in quadruplets in 24 well plate overnight. After 24 h the cells were exposed to either selinexor, DEX or EVER at indicated doses in quadruplets for additional 72 h. At the end of the treatment period individual treatment quadruplet was pooled into one and total RNA was isolated using Trizol (Life Technologies) in accordance with the manufacturer's described protocol. Briefly 1 ml of Trizol was mixed with 200 µl of chloroform and centrifuged at 12,000 × g for 15 min at room temp. The upper aqueous phase was mixed with equal amount of isopropanol and centrifuged at a speed of 12,000 × g for 10 min. The resulting pellet was washed with 80% EtOH; air dried and was eluted with nuclease free water. We utilized a high capacity cDNA reverse transcription Kit (Applied BioSystems, Foster City, CA, USA) to measure them RNA level. Approximately 1 µg of RNAs from the different samples was reverse transcribed using 5.8 µl of master mix. The mixture was incubated for 10 min at 25 °C, then 37 °C for another 120 min, and finally 85 °C for 5 min. Quantitative-PCR was performed in triplicate on all samples using SYBR Green PCR Master mix (Life Technologies), cDNA sample. The following primers were used: Akt (ACTA1_F:ACAATGTGCGACGAAGACGA; ACTA1_R:GACCCATACCGACCATGACG), mTOR (mTOR_F:TTCCGACCTTCTGCCTTCAC; mTOR_R:CCACAGAAAGTAGCCCCAGG) and raptor (RAPTOR_F:GACCTCGTGAAGGACAACGG; RAPTOR_R:CTTCCTGCCCCGTGTGATAG). GAPDH (GAPDH-96F:CCACATCGCTCAGACACCAT; GAPDH-96R:ACCAGAGTTAAAAGCAGCCCT) was used to normalize the expression level of genes. Graphs were plotted using Graph Pad Prism software.

### Development of animal xenografts and pre-clinical efficacy trial

Mouse xenografts were established as described previously [19] The maximum tolerated dose (MTD) of selinexor in severe combined immunodeficient (ICR-SCID) mice was determined to be 15 mg/kg orally (every other day × 3/week) or with other SINE analogs as indicated. Mice were treated with selinexor orally at doses of 15 mg/kg every other day 3 days a week for three weeks. The CHOP regimen was used as the positive control at the MTD, as described previously [19]. Mice in the control and SINE compound treated groups were followed for measurement of subcutaneous tumors, changes in body weight, and other side effects of the drugs. Tumors were measured twice weekly. Tumor weight (mg) was calculated using the formula: (*A* × *B^2^*)/2, where *A* and *B* are the tumor length and width (in mm), respectively. To avoid discomfort and in keeping with our IACUC procedures, animals were euthanized when their total tumor burden reached 2000 mg. All studies involving mice were done under Animal Investigation Committee-approved protocols.

## Results

### Selinexor enhances the activity of DEX and EVER in WSU-DLCL2 and WSU-FSCCL NHL cell lines

**Fig. 1:**
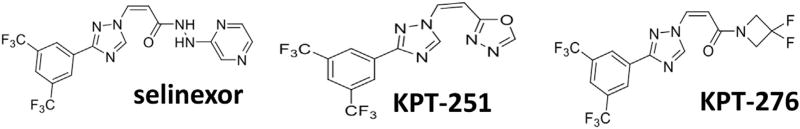
Structures of the Specific Inhibitors of Nuclear Export (SINE) used in this study.

**Fig. 2:**
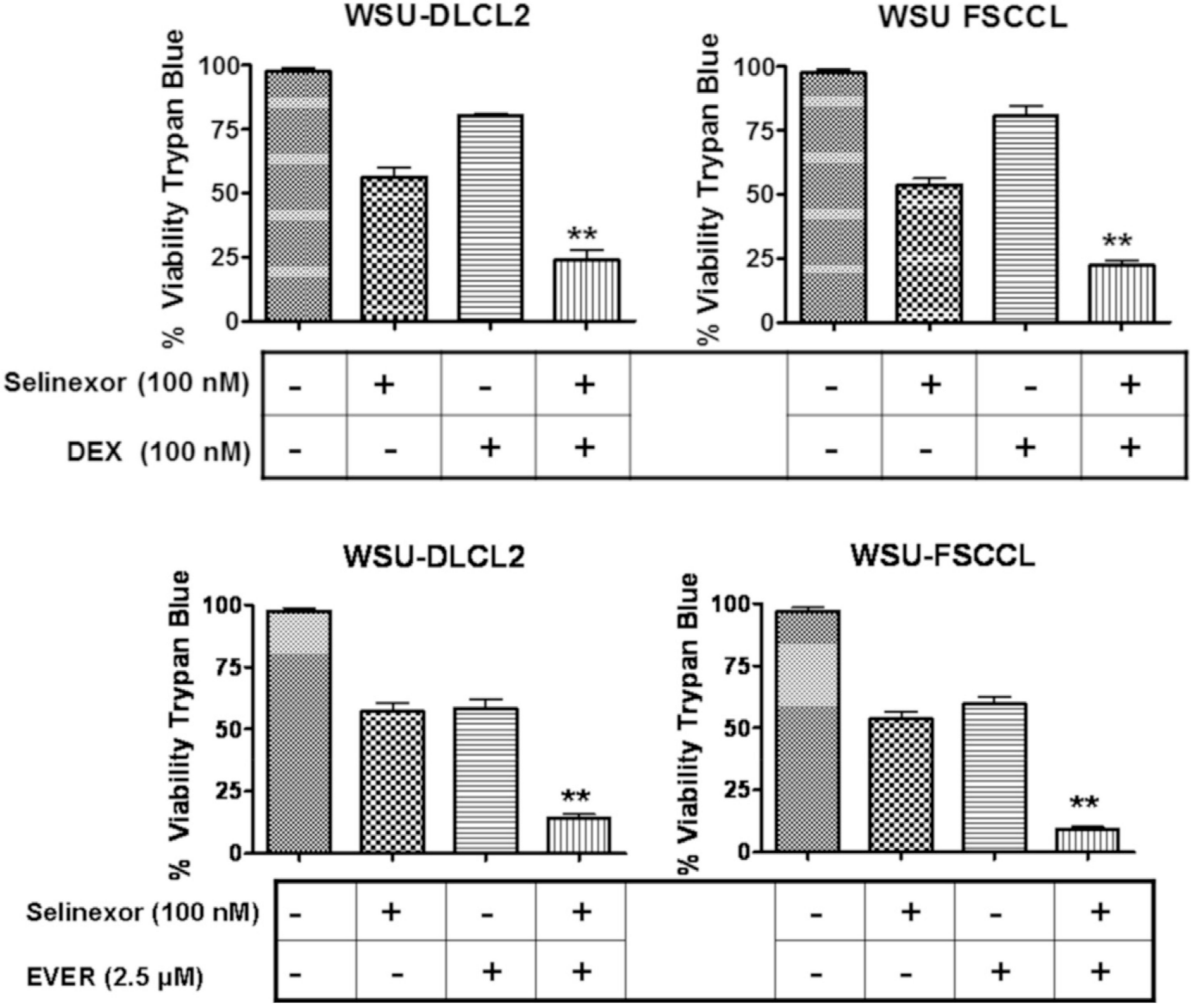
Selinexor synergizes with dexamethasone and everolimus leading to superior cytotoxicity in NHL. 1 × 10^6^ WSU-FSCCL or WSU-DLCL2 cells were seeded in triplicate in 24 well plates and incubated with 100 nM selinexor (SEL) or 100 nM dexamethasone (DEX) or 1.25 µM everolimus (EVER), each drug alone, SEL + DEX or SEL + EVER for 72 h. Resulting cell viability was determined using trypan blue staining [trypan blue (0.4%), Sigma Chemical Co. St. Louis, MO, USA] and cell counting. Data representative of three independent experiments with three replicates per concentration. *p < 0.05 and **p < 0.01.

The structures of SINE compounds used in this study are given in Fig. 1. Our laboratory has extensively studied selinexor activity against DLBCL [15]. Table 1 lists the different IC_50_s of selinexor (Trypan Blue exclusion assay) against a number of well characterized DLBCL cell lines. DEX is being employed extensively in the clinic either as single agent or in combination therapies with other chemotherapeutics for many hematological malignancies including DLBCL. Similarly, the targeted mTOR inhibitor, everolimus (EVER), is also gaining traction in combination regimens for different hematological malignancies [20]. Given that DEX targets cell survival signals through inhibition of NF-κB [21] which in turn promotes mTOR signaling [22], we explored the consequence of the combination against two well characterized NHL cell lines, WSU-DLCL2 and WSU-FSCCL. As can be seen from Fig. 2 combination of selinexor with DEX or EVER resulted in statistically significant (p < 0.01) enhancement of cytotoxicity in both these cell lines. These data show the potential for synergistic mechanisms of action with these proposed combinations.

### Combination of selinexor with DEX or EVER increases apoptosis when compared to single agents alone

**Fig. 3:**
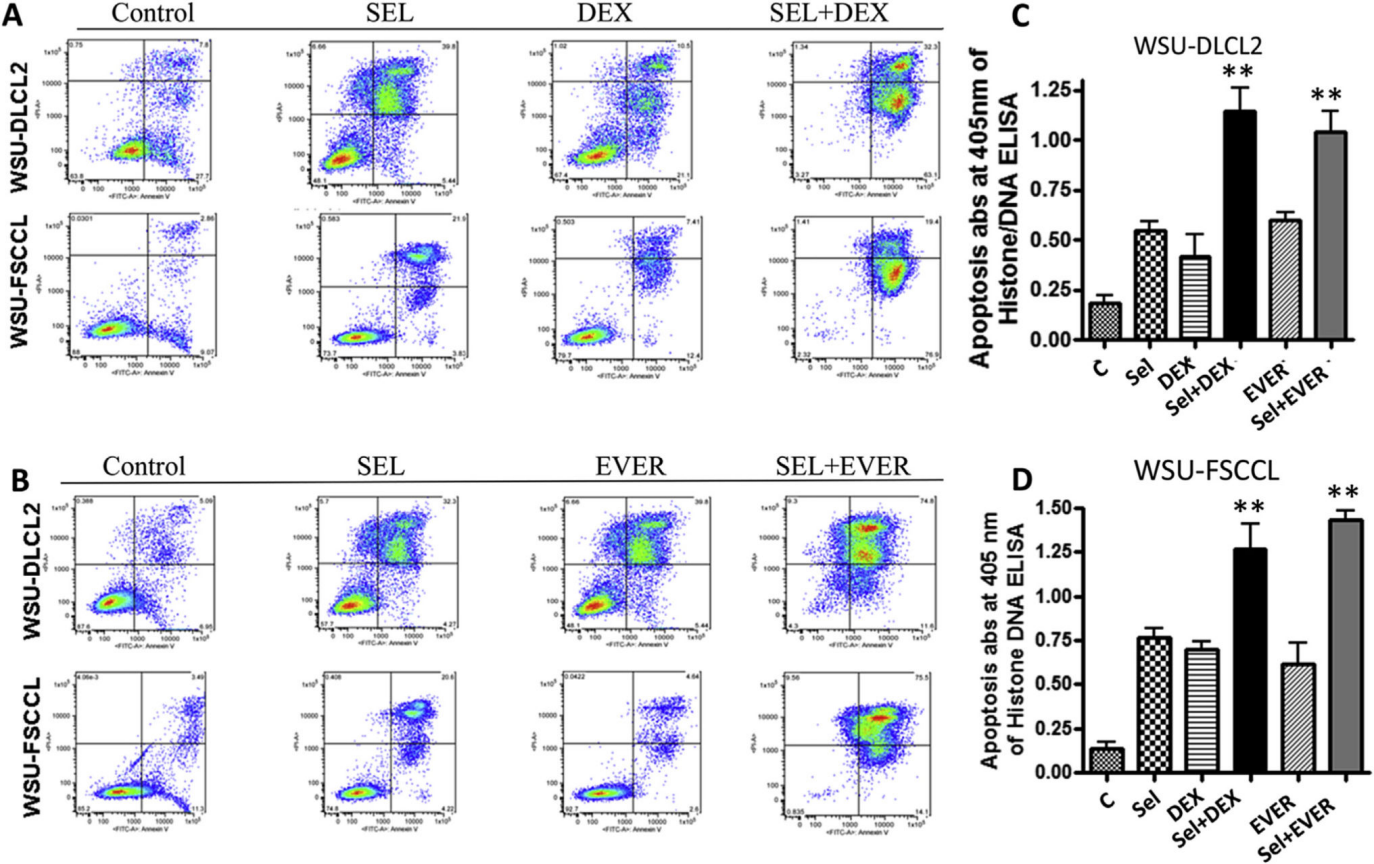
Apoptosis analysis. [A and B] 1 × 10^6^ cells were seeded per well in triplicate in 24 well plates and exposed to 100 nM selinexor (SEL) or 100 nM DEX or 1.25 µM everolimus (EVER), each drug alone, SEL + DEX or SEL + EVER for 72 h. After the treatment period was over, the cells were centrifuged at 3000 rpm and the media was removed. The cell pellet was dissolved in 500 µL of Annexin binding buffer and mixed with 5 µL of Annexin and 5 µL of propidium iodide reagent (Biovision, USA). **[C and D]** Apoptosis analysis using Histone DNA ELISA under similar treatment conditions (Annexin V FITC assay was performed according method supplied by the manufacturer Roche Death assay kit (Roche Cat #11774425001). Results are representative of three independent experiments. *p < 0.05 and **p < 0.01 when compared to single agent treatment.

Having demonstrated the enhancement in cytotoxicity of the combination using Trypan Blue, we evaluated the apoptotic potential of these combinations using Annexin V FITC assays. As predicted the decrease in viability was concurrent with statistically significant (p < 0.01) increase in apoptosis in the DEX or EVER combination with selinexor compared to single agents alone in both NHL cell lines (Fig. 3A and B). For additional proof of these synergies, we evaluated the combinations using a histone DNA/ELISA assay. In accordance with Annexin V staining, the histone DNA/ELISA assay demonstrated increased apoptosis in the selinexor-DEX or selinexor-EVER combination compared to single agent treatments (p < 0.01) (Fig. 3C and D). Considering the combination was effective in WSU-DLCL2 cells, a Myc, BCL-2/6 double hit model, these results provide strong indication that the combinations may be viable therapeutic options regardless of the mutational status of Myc and BCL-2/6 in therapy resistant NHL.

### Mechanistic insights into selinexor-DEX and selinexor-EVER synergy

**Fig. 4:**
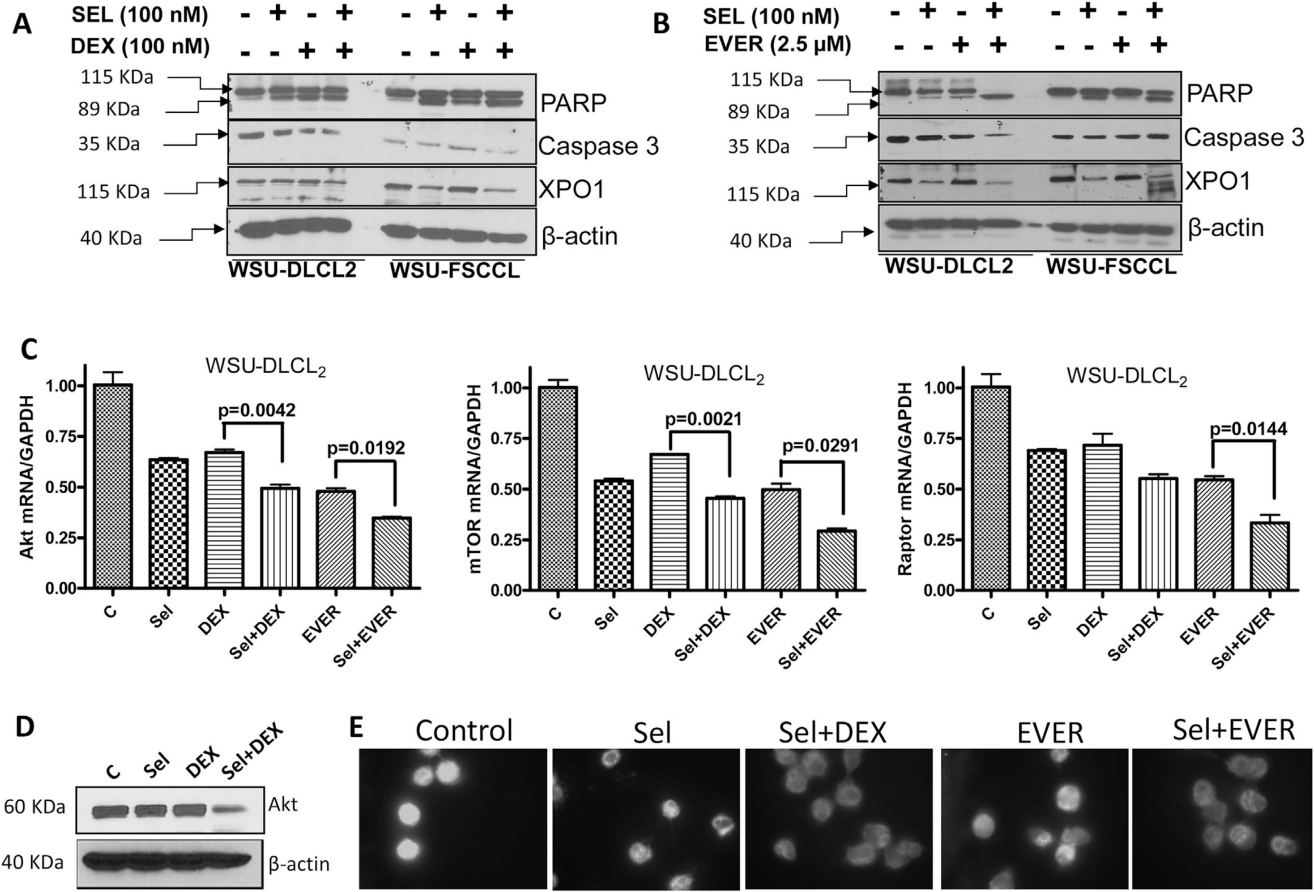
Molecular analysis of SEL-DEX and SEL-EVER combination. WSU-DLCL2 or WSU-FSCCL were grown at a density of 1 × 10^6^ per well in six well plates and exposed to indicated concentrations of drugs for 72 h followed by protein isolation and western blotting. [**A and B**] Results showing enhanced PARP cleavage, full length caspase 3 reduction and decrease in XPO1 expression for combination treatments compared to single agents. β-actin was used as loading control. Blots are representative of three independent experiments. **[C]** WSU-DLCL2 cells were grown in 24 well plates at a density of 200,000 cells per well overnight. The next day cells were exposed to either DMSO, selinexor, DEX or their combination for additional 72 h in quadruplets. RNA was isolated and RT-PCR was performed according to procedure described in methods section. Relative expression of mRNAs was analyzed by utilizing the Ct method and was normalized to GAPDH. p Values were calculated using Graph Pad Prism software. **[D]** WSU-DLCL2 cells were grown in 24 well plates at a density of 200,000 cells per well overnight. The next day cells were exposed to either DMSO, selinexor, DEX or their combination for additional 72 h in quadruplets. Protein isolation and western blotting was performed according to procedure described in methods section. Membranes were probed for Akt (Cell signaling Danvers, MA USA). The blots were re-probed for β-actin as loading control. **[E]** WSU-DLCL2 cells were grown at a density of 2 × 10^3^ per well in duplicate in 24 well plates and exposed to vehicle or selinexor (100 nM) + DEX (100 nM) or selinexor (100 nM) + Ever (1.25 µM) for 24 h. At the end of the treatment period, cells were spun down on a glass slide using cytospin (3000 rpm). The slides were subjected to immunofluorescence assay with p65 antibody (Santa Cruz). Briefly, the slides were fixed with 10% paraformaldehyde for 15 min. Followed by 3 washes in TBST (5 min each) and permeabilization in 0.5% Triton for 10 min. After additional 3 washes (in PBST 5 min each), the slides were blocked in 0.2% BSA and probed with primary and secondary antibodies according to our previously published methods [31]. The slides were dried and mounting medium was added to it and covered with a coverslip and were analyzed under an inverted fluorescent microscope. A total of two independent experiments were performed.

In order to evaluate the molecular mechanism underlying these efficacious combinations, we evaluated several known signaling pathways. When comparing to single agent treatment, we observed significant enhancement in PARP cleavage by the combination treatment of selinexor-DEX or selinexor-EVER (Fig. 4A and B). Additionally, we observed reduction in the expression of full length caspase-3 in the combination treatments compared to single agents in both of these cell lines. Interestingly, we observed a more substantial decrease in the selinexor target XPO1 in the combination treatment groups when compared to the single agents. These results suggest that the combination of selinexor with either of these two agents may potentially be a stronger inhibitor of XPO1 activity. The impact of the combination on mTOR signaling was also evaluated using RT-PCR. As can be seen from the results of Fig. 4C, compared to single agent treatment, the combination of selinexor with DEX or EVER resulted in statistically significant and superior inhibition of Akt, mTOR and rictor mRNA levels (p < 0.001). Western blotting data supported RT-PCR results where we also observed reduction in Akt levels in the combination treatment (Fig. 4D).

In addition, we tested the impact of these combinations using immunofluorescence assay. As can be seen from the results presented in Fig. 4E, the combination treatment caused marked reduction in the nuclear expression of DEX target p65 when compared to either untreated control cells or single agent treatments. Collectively, these results clearly show that there is an underlying molecular synergy between selinexor-DEX or selinexor-EVER that leads to superior anti-lymphoma activity.

### SINE compounds demonstrated comparable activity to CHOP in subcutaneous and systemic NHL xenografts in mice

**Fig. 5:**
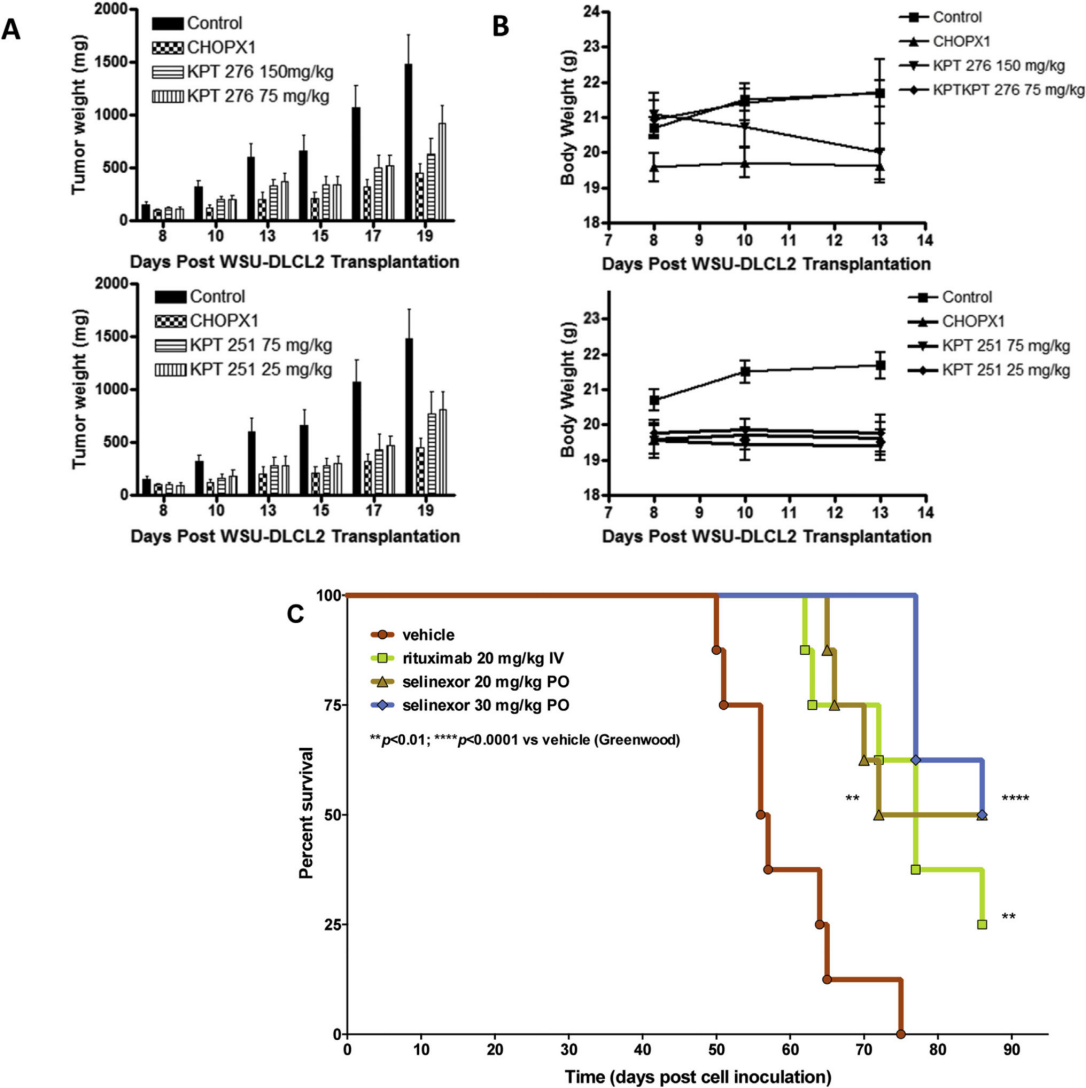
Equivalent *in vivo* efficacy of single agent selinexor vs rituximab or CHOP. [**A** and **B**] Xenograft model of DLBCL. SINE Compounds KPT-251 and KPT-276 were administered sc and po, respectively, in cycles of once daily for ten consecutive days with a one day break prior to start of a new cycle. Cyclophosphamide, doxorubicin and vincristine (CHO) was administered once IV at MTD and prednisolone (P) was administered po QDX5. **[C]** 10 × 10^6^ WSU-FSCCL follicular lymphoma cells were injected IV in the tail veins of ICR-SCID mice. After 1 week of inoculation, the mice were randomly divided into different group (n = 6) and vehicle or drug treatments were started one week later as indicated.

In earlier studies we confirmed the activity of selinexor analogs such as KPT-185, KPT-251 and KPT-276 against NHL models *in vitro* and *in vivo* [15]. Building on these findings, we compared the efficacy of different selinexor analogs to CHOP and rituximab (R). Compared to CHOP (used at standard doses), KPT-276 (75 and 150 mg/kg) and KPT-251 (25 and 50 mg/kg) showed similar or enhanced anti-tumor potential against WSU-DLCL2 xenograft (Fig. 5A). The doses were well tolerated by mice and the minimal loss in body weight was recovered once the treatment was stopped (Fig. 5B). More striking results were observed in the systemic model. Compared to rituximab, we observed statistically significant enhancement in survival of the selinexor treated animals harboring the WSU-FSCCL model (Fig. 5C). These findings support that (a) selinexor has activity comparable to the standard of care (e.g. CHOP) and; (b) selinexor has to some extent better tolerability when compared to CHOP or related toxic regimens.

### SINE compounds synergize with DEX and EVER in sub-cutaneous and systemic models of NHL

**Fig. 6:**
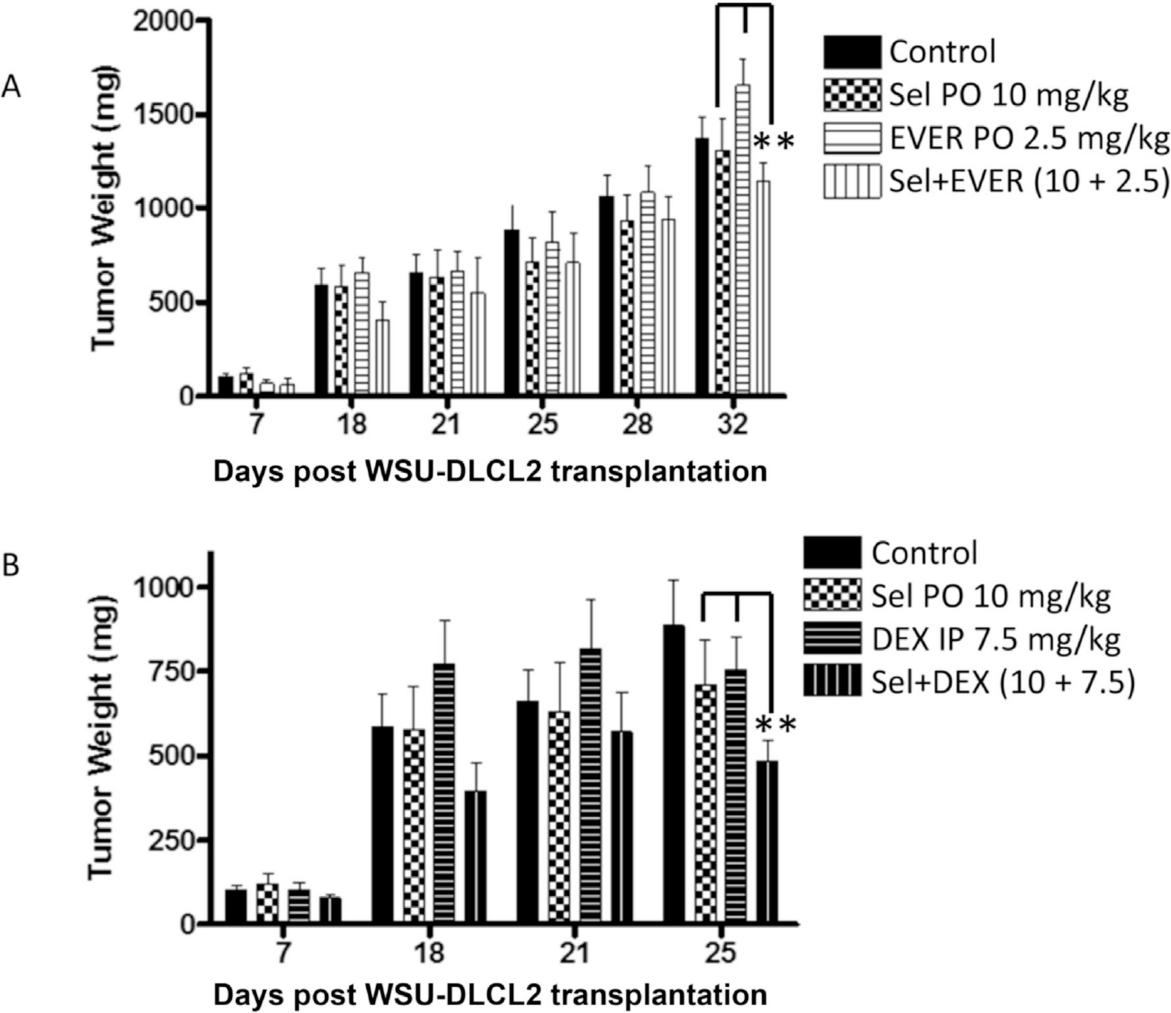
Selinexor at sub-optimal doses enhances the activity of DEX or EVER in subcutaneous DLBCL xenografts. WSU-DLCL2 xenograft were established as described above. Drugs were administered at indicated doses 5 days a week for three weeks. [**A**] SEL + EVER and [**B**] SEL + DEX combination (study continuing beyond 25 days). selinexor (orally 10 mg/kg every other day for three weeks); EVER (2.5 mg/kg orally); combination of selinexor (orally 10 mg/kg every other day for three weeks); EVER (2.5 mg/kg orally); DEX (7.5 mg/kg ip once a week for three weeks) and selinexor (orally 10 mg/kg every other day for three weeks) + DEX (7.5 mg/kg ip once a week for three weeks). **p < 0.01 when compared to single agent treatments.

**Fig. 7:**
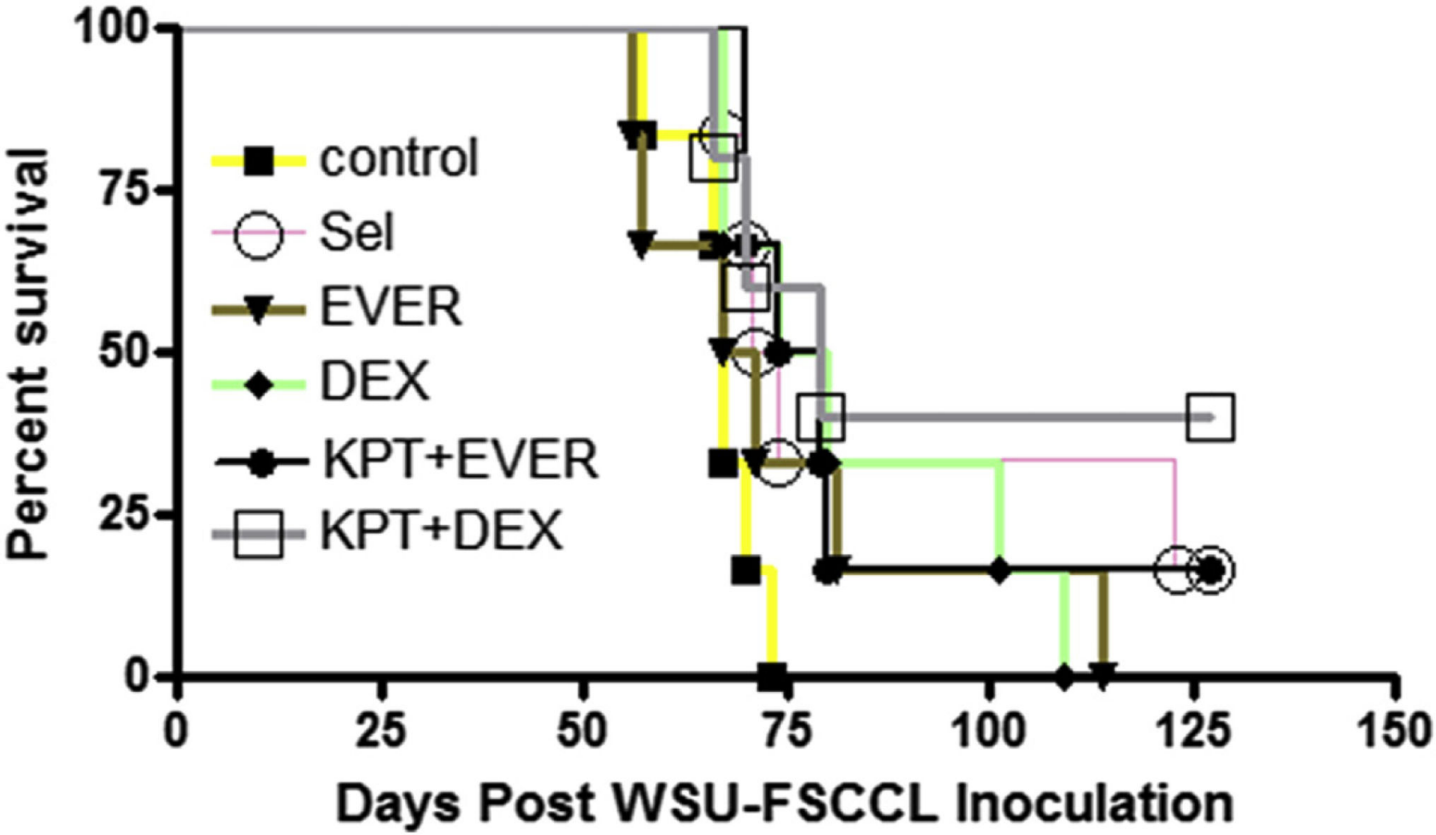
Evaluation of selinexor-DEX and selinexor-EVER combination in systemic (disseminated) model of NHL. 10 × 10^6^ WSU-FSCCL cells were injected via tail vain. One week after cell injection, mice were randomly divided in different treatment groups (n = 6) and treated with selinexor (orally 10 mg/kg every other day for three weeks); EVER (2.5 mg/kg orally); combination of selinexor (orally 10 mg/kg every other day for three weeks); EVER (2.5 mg/kg orally); DEX (7.5 mg/kg ip once a week for three weeks) and selinexor (orally 10 mg/kg every other day for three weeks) + DEX (7.5 mg/ kg ip once a week for three weeks). The mice were followed for 90 days (till death occurs). Color coded lines to differentiate the differences between each treatment groups. (For interpretation of the references to colour in this figure legend, the reader is referred to the web version of this article).

Having demonstrated single agent activity of SINE compounds in multiple NHL xenograft models, we sought to evaluate the efficacy and tolerability of the selinexor-DEX and selinexor-EVER combinations in WSU-DLCL2 sub-cutaneous and WSU-FSCCL systemic model of NHL. Oral administration of selinexor at a submaximum tolerated dose, DEX or EVER demonstrated limited reduction of WSU-DLCL2 tumor xenograft (Fig. 6A and B). However, the combinations showed statistically significant (p < 0.01) tumor inhibition when compared to single agents alone. In the disseminated model similar results were observed (Fig. 7). In DEX or EVER single agent treatment, extension in survival (days) was observed when compared to control. However, in the selinexor-DEX combination, two mice remained alive beyond 100 days after all the other mice had died in the other groups. Taken together these results extend the *in vitro* synergy of the selinexor combinations to *in vivo* and increase the potential for use in a human clinical trial.

## Discussion

In this paper we demonstrated that the clinical SINE compound, selinexor, combined with dexamethasone (DEX) or everolimus (EVER) lead to enhanced *in vitro* cytotoxicity in NHL cells when compared to single agent treatment alone. We also demonstrated that these combinations have improved anti-lymphoma activity in a sub-cutaneous as well as disseminated xenograft model of NHL. Selinexor, currently in Phase I and II clinical trials for the treatment of various hematological malignancies, has shown tolerability and activity as a single agent in patients with NHL. Our pre-clinical combination studies provide strong rationale for testing in clinical trials of NHL.

Gene-expression analysis have increased our understanding of the molecular basis of chemotherapy resistance and identified rational targets for drug interventions to prevent and treat relapsed/refractory diffuse large B-cell lymphoma [23]. Acquisition of drug resistance in lymphoma is in part driven by the inherent genetic heterogeneity and instability of tumor cells [5]. PI3K/AKT/mTOR is one of the most frequently deregulated cell survival pathways in cancer (14). In NHL, aberrant activation of this pathway involves diverse mechanisms, including loss of the tumor suppressor PTEN (through mutation or inactivation), PI3Ka mutations, PI3Kd overexpression/activation, and BCR receptor activation [15–17]. Activation of PI3K leads to upregulation of multiple downstream effectors that include the AKT–mTOR axis and plays a critical role in diverse cell processes, such as growth, survival, metabolism, and autophagy [18]. Therefore, the PI3K-AKT-mTOR axis is a critical pathway in NHL disease development requiring renewed focus and attention.

XPO1 is a member of the karyopherin β superfamily of nuclear transport receptors, recognizing proteins bearing a leucine-rich nuclear export sequence (NES) [24]. There are seven known nuclear export proteins, but only XPO1 mediates the export of nearly all major TSPs out of the nucleus. Nuclear exclusion of p53, FOXO, p27, and others by XPO1 renders cancer cells resistant to apoptosis by different therapies [25]. Specifically, the mTOR protein possesses a NES and is an export target of XPO1. Forced nuclear retention of TSPs by inhibition of XPO1 (without affecting their nuclear import) prevents proteasome-mediated degradation in the cytoplasm and leads to restoration of their tumor-suppressing activities in the nucleus [9] Nuclear localization with functional activation of TSPs through SINE compound treatment of cells leads to selective elimination of tumor cells. Inhibition of XPO1 is one approach to restore nuclear localization and activation of multiple TSPs, allowing them to function properly and induce cancer-specific apoptosis.

The potent XPO1 inhibitor, Leptomycin B (LMB), was first isolated from Streptomyces as an anti-fungal compound [26]. LMB also demonstrated considerable anti-tumor activity *in vitro* and in animal studies. Because of this activity, LMB (elastocin) was used in a single Phase I human trial [27]. However, LMB displayed considerable toxicity, which lead to its withdrawal from the clinic and restricted its use to an *in vitro* tool compound. Recently, Karyopharm Therapeutics has developed a novel class of XPO1 inhibitors called Selective Inhibitor of Nuclear Export (SINE) compounds [28]. Biochemical as well as CRISPR/Cas9 genome editing studies have confirmed the specificity of these compounds as slowly reversible covalent binders of the cysteine 528 residue of XPO1 located in NES cargo binding pocket [12].

In our previous studies we demonstrated that the single agent anti-tumor activity of selinexor and its analogs against NHL models both *in vitro* and *in vivo* [15]. We mechanistically deduced that the nuclear retention of TSPs (i.e. p53, p63, FOXO, p27 and p73) was critical for SINE compound activity in NHL [15]. Our studies as well as data from multiple other groups led to the clinical evaluation of selinexor in Phase I clinical trials for the treatment of hematological and solid malignancies. In these trials selinexor has shown tolerability and single agent anti-lymphoma activity in patients that have failed all other standard of care therapies. As an extension of our previous work and combination data from the current clinical trials, we evaluated the combination of selinexor with approved NHL clinical therapeutics, including dexamethasone (DEX) and Everolimus (EVER). Unlike our previous studies, where we used a maximum tolerated dose of selinexor (15 mg/kg or higher for other related analogs KPT-251 and KPT-276), the doses used in this study were sub-optimal (5 mg/kg) yet better tolerated. Similarly, dexamethasone and EVER were used at sub-optimal doses in their respective combinations with selinexor. From our encouraging *in vitro* and *in vivo* results, we propose that low doses of either EVER or Dex with a lower selinexor dose are sufficient to induce antitumor response and have improved tolerability. This certainly circumvents the toxicity related issues associated with these two commonly used drugs. The pre-clinical benefit of combining these agents *in vitro* as well as the benefit demonstrated in multiple *in vivo* models strengthens the rationale for using selinexor-DEX or selinexor-EVER combination in Phase II clinical trials for NHL.

Supporting the translational potential of our proposed studies, selinexor is being tested in 48 different Phase I and II human clinical trials (clinicaltrials.gov: KPT-330) [29]. The selinexor Phase I clinical study of hematological cancers which included a significant number of NHL patients has demonstrated suitable tolerability and therapeutic response (NCT01607892). In this study the patients with relapsed and/or refractory NHL, including DLBCL, had a disease control rate of 74% across all doses of selinexor with an overall response rate (ORR; partial response or better) of 28% [30]. In our *in vitro* studies we observed potent anti-tumor activity of selinexor in MYC and BCL2/6 “double-hit” DLBCL, while the clinical responses were observed across all subtypes of NHL independent of genetic abnormalities. Currently, a Phase I clinical study is evaluating selinexor in combination with Ibrutinib (a Bruton's Tyrosine Kinase inhibitor) for the treatment of patients with relapsed or refractory chronic lymphocytic leukemia (CLL) or aggressive NHL (NCT02303392). These current trials as well as our results from the present studies certainly build a rationale for testing additional selinexor combinations for the treatment of patients with therapy resistant NHL.

## Figures and Tables

**Table 1:** Selinexor shows anti-lymphoma activity against a spectrum of DLBCL cell lines irrespective of the BCL2/6 or Myc mutation status.

DLBCL cell line	Selinexor IC_50_ (µM)
RL	0.020
OCILY3	0.050
A3/KAW	0.057
OCILY19	0.063
SUDHL5	0.070
SUDHL8	0.096
DOHH2^a^	0.120
WSU-DLCL2^a^	0.150
SUDHL6	0.29
TOLEDO	0.44
DB	0.55
WSU-FSCCL	0.110

a Double hit DLBCL cell models. IC_50_s calculated using Trypan Blue exclusion test at 72 h treatment.
